# Research on Rapid Detection Methods of Tea Pigments Content During Rolling of Black Tea Based on Machine Vision Technology

**DOI:** 10.3390/foods13233718

**Published:** 2024-11-21

**Authors:** Hanting Zou, Tianmeng Lan, Yongwen Jiang, Xiao-Lan Yu, Haibo Yuan

**Affiliations:** Tea Research Institute, The Chinese Academy of Agricultural Sciences, Hangzhou 310008, China; 13500436167@163.com (H.Z.); ww98317@163.com (T.L.); jiangyw@tricaas.com (Y.J.); yuxiaolan@tricaas.com (X.-L.Y.)

**Keywords:** black tea rolling, color features, machine vision, machine learning

## Abstract

As a crucial stage in the processing of black tea, rolling plays a significant role in both the color transformation and the quality development of the tea. In this process, the production of theaflavins, thearubigins, and theabrownins is a primary factor contributing to the alteration in color of rolled leaves. Herein, tea pigments are selected as the key quality indicators during rolling of black tea, aiming to establish rapid detection methods for them. A machine vision system is employed to extract nine color feature variables from the images of samples subjected to varying rolling times. Then, the tea pigment content in the corresponding samples is determined using a UV-visible spectrophotometer. In the meantime, the correlation between color variables and tea pigments is discussed. Additionally, Z-score and PCA are used to eliminate the magnitude difference and redundant information in original data. Finally, the quantitative prediction models of tea pigments based on the images’ color features are established by using PLSR, SVR, and ELM. The data show that the Z-score–PCA–ELM model has the best prediction effect for tea pigments. The Rp values for the model prediction sets are all over 0.96, and the RPD values are all greater than 3.50. In this study, rapid determination methods for tea pigments during rolling of black tea are established. These methods offer significant technical support for the digital production of black tea.

## 1. Introduction

As one of the most popular types of tea on the market, black tea is favored by consumers both domestically and internationally due to its distinctive flavor and functional components [1]. Compared to other types of tea, the formation of the flavor quality of black tea, as a fully fermented type of tea, is closely related to the processing technology used. The postharvest processing of black tea can be broadly classified into four stages: withering, rolling, fermentation, and drying [2]. Rolling not only significantly affects the appearance and texture of the leaves, but also lays a chemical substance basis for the subsequent fermentation stage. Simply speaking, rolling refers to the mechanical process of shaping the withered leaves into strips [3]. Under the action of external mechanical force, the cellular tissues of tea leaves are destroyed. Consequently, this damage also facilitates the release of intracellular substances, promoting polyphenols to interact with enzymes [4]. These polyphenols undergo oxidation reactions, resulting in the formation of pigment compounds such as theaflavins (TFs), thearubigins (TRs), and theabrownins (TBs) [5]. These pigment compounds are not only pivotal in the flavor of black tea, but also play a direct role in influencing the color changes observed in rolled leaves. Despite the fact that traditional stoichiometric methods can determine the content of these chemical components accurately, there is a lag in the collection, storage, transportation, and detection of the samples. This is not conducive to the efficient industrial production of black tea. At present, there are many non-destructive testing methods for tea [6,7,8], but there is still a lack of online testing technology for employment in the rolling process of black tea. Therefore, the real-time and rapid detection of tea pigments in rolled leaves is of great significance in black tea production and processing.

In recent research surrounding tea and other agricultural product testing, non-destructive testing technology has been widely used [9,10,11]. During tea processing, the use of sensors and other instruments to monitor the content of key substances online and effectively classify the processing degrees of samples are the goals of intelligent processing technology. As a kind of bionic vision technology, machine vision can obtain the color features and other information of samples through industrial cameras in real-time [12]. Unlike near-infrared spectroscopy, machine vision can indirectly associate certain chemical components with color changes in the appearance of samples. At present, it has been used in the detection of theaflavins and chlorophyll during the fermentation stage of black tea [13].

To summarize, machine vision technology is employed in this study to capture images of rolled leaves at various rolling times. Meanwhile, a rapid non-destructive detection method for tea pigments in the rolling process of black tea is established by combining image color characteristics and chemometrics methods. The color feature variables extracted from the images include RGB, HSV, and La*b*. Pearson correlation analysis is used to express the response intensity of color feature variables to tea pigments content. In addition, the application of Z-score and PCA effectively eliminates the information interference between color feature variables. Finally, a quantitative prediction model with strong generalization capabilities is established based on a linear model, nonlinear model, and neural network model. The analysis and comparison of various algorithm models is instrumental in developing effective methods for the rapid detection of tea pigments during the rolling of black tea.

## 2. Materials and Methods

### 2.1. Chemicals and Reagents

Ethyl acetate, n-butanol, oxalic acid, sodium bicarbonate, and ethanol were purchased from Aladdin Biochemical Technology Co., Ltd. (Shanghai, China). Pure water was prepared using a Pure Water System.

### 2.2. Sample Preparation

The tea variety selected in this study was Fuding Dabai, and the picking tenderness of fresh leaves was one bud and two leaves. The moisture content of the sample was determined by a moisture analyzer (MA35M-000230V1, Sartorious, Göttingen, Germany) during processing. The moisture content of fresh leaves was about 77.53%. First, the fresh leaves were evenly spread in a withering trough, maintained at a thickness of about 2–3 cm. At room temperature of about 24 °C and relative humidity of about 60%, the withering process ended after about 12 h. The moisture content of samples determined after withering was about 61.08%. Soon afterwards, the samples were evenly divided into nine groups of the same quality. They were then rolled in batches according to the set sampling time.

Rolling machines (6CR-45, Xiangfeng Machinery Co., Ltd., Tongxiang, China) were used, and the rolling parameters were set as follows: no-pressure for 20 min, light-pressure rolling for 20 min, heavy-pressure rolling for 10 min, light-pressure rolling for 10 min, heavy-pressure rolling for 10 min, and light-pressure rolling for a further 10 min. To collect samples of over-rolling, the final rolling time was extended by another 10 min, for a total of 90 min. From the end of withering to the end of rolling, sampling points were set every 10 min, for a total of 10 sampling points. After each batch of samples reached the corresponding rolling time, all samples were taken out. All samples taken each time were evenly divided into eight groups for data collection and preservation. The next day, the same operation was repeated. As a result, a total of 150 samples of rolled leaves were collected throughout the entire process.

### 2.3. Determination of Tea Pigments Content

The content of theaflavins (TFs), thearubigins (TRs), and theabrownins (TBs) was determined by referring to the system analysis method [14] and through quantitative analysis using a UV-visible spectrophotometer (P7, MAPADA, Shanghai, China). First, NaHCO_3_ (2.5%) solution and saturated oxalic acid solution were prepared. A total of 3 g of tea sample was weighed out and transferred to a 250 mL conical bottle, and 125 mL boiling water was added. The tea sample was then soaked in a boiling water bath for 10 min and shaken intermittently. After the extraction, the solution was strained immediately with filter cloth, and the filtrate was allowed to cool. This filtrate was marked as the test solution.

The test solution was shaken well. Following this, 30 mL of it was removed with a pipette gun and placed into a separating funnel, to which 30 mL of ethyl acetate was added. Then, an oscillator (185/min) was used to shake the mixture for five minutes. Once standing and layering had occurred, the lower water layer was released, and the ethyl acetate layer above was poured out. A 2 mL extract of the ethyl acetate layer was diluted to a total volume of 25 mL using 95% ethanol. The absorbance values of the solution were recorded as EA.

A 2 mL extract of the water layer was diluted to a total volume of 25 mL using 2 mL saturated oxalic acid, 6 mL pure water, and 95% ethanol. The absorbance values of the solution were recorded as ED.

Then, 15 mL of the ethyl acetate layer was taken, transferred to the separation funnel, and 15 mL of NaHCO_3_ (2.5%) solution was added. The mixture was shaken for 30 s with the oscillator. Following the completion of layering, the lower sodium bicarbonate layer was discarded, and the ethyl acetate layer above was saved. A total of 4 mL of the ethyl acetate layer was diluted to a total volume of 25 mL using 95% ethanol. The absorbance values of the solution were recorded as EC.

Finally, 15 mL of test solution was taken and transferred to the separation funnel. Then, 15 mL of n-butanol was added, and the mixture was shaken with the oscillator for 3 min. Once layering had occurred, the lower water layer was saved. A total of 2 mL of this water layer was diluted to a total volume of 25 mL using 2 mL saturated oxalic acid, 6 mL pure water, and 95% ethanol. The absorbance values of the solution were recorded as EB.

The values of EA, EB, EC, and ED were measured at 380 nm with 95% ethanol as the control [15]. The formulas for calculating the content of each component are as follows:

TFs (%) = EC × 2.25(1)

TRs (%) = 7.06 × (2 × EA + 2 × ED − EC − 2 × EB)(2)

TBs (%) = 2 × EB × 7.06(3)

### 2.4. Image Information Acquisition

**Figure 1:**
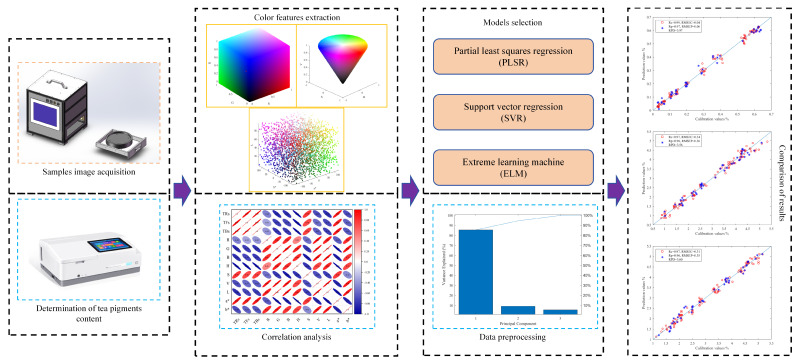
Flow chart of the experiment.

In this research, a custom-designed machine vision system was employed for data acquisition. The machine vision system is an industrial integrated machine composed of a high fidelity industrial camera (MV-CS020-10GMGC, Hikvision Digital Technology Co., Ltd., Hangzhou, China), a professional industrial lens, a dome shadowless light source (pure white, LED), a control panel, visual inspection software, and other core components. The camera has a resolution of 20 million pixels. The color array camera was used to shoot the tea samples under the dome light source, and the closed equipment box was used to ensure that the detection was not affected by the ambient light and maintain detection stability. The distance between the camera lens and the sample was about 6 cm, and the exposure time was 0.08 ms. During the experiment, the sample was evenly spread in the preset shooting area, and the thickness of it was controlled at about 2 cm. In order to make the data obtained more reliable, two to three different images were taken for each group of samples. These images were used to calculate the average values of color feature variables. To sum up, the experimental process is shown in Figure 1.

### 2.5. Color Features Information Extraction

Here, the machine vision system was used to automatically select a pixel region in the shooting area of rolled leaves, and this part of the region was regarded as the region of interest (ROI) [16]. Concurrently, the machine learning algorithms were used to extract color feature variables in the ROI. The average values of each color feature variable in the ROI were calculated, and they were used to represent the color feature information of each group of samples. As a result, each image was converted into nine color feature variables, including three color models, namely, RGB, HSV, and La*b*.

RGB is not only widely used in mobile phones, monitors, and other devices, but is also crucial in the research of graphics processing [17]. It consists of R, G, and B values (R: red, G: green, B: blue). Therefore, various other visible colors can be produced by changing the combination of R, G, and B values to different intensities. However, in some cases, it is found that this color model does not fully meet needs. For example, in terms of color perception, people’s color cognition does not only rely on red, green, and blue. Therefore, in order to simulate human perception of color more accurately, HSV was introduced. It is composed of H, S, and V values (H: hue, S: saturation, V: brightness) [18]. Moreover, La*b* consists of L, a*, and b* values (L: brightness, a*: red-green degree, b*: yellow-blue degree), and is also widely used in color science [19]. In summary, the three color models of RGB, HSV, and La*b* have their own advantages and disadvantages, and are suitable for different scenes and needs. RGB plays a dominant role in digital image processing, while HSV is closer to human color perception.

### 2.6. Data Set Partitioning and Preprocessing

The random sampling method was used to divide the data set [20], and the ratio of training set to prediction set was set at 7:3. First, all the sample data were arranged in ascending order according to the corresponding tea pigments content. From top to bottom, each of the 10 sample data sets was divided into a group. In each group, random sampling was carried out, in which three groups of sample data were used as prediction set data, and the remaining seven groups of sample data were used as training set data. After partitioning, the obtained training set data contained 105 groups of sample data, while the prediction set data contained 45 groups of sample data. The color feature information in the images was derived from three distinct color models. The methods for calculating color features varied among these models, leading to differences in values. In order to eliminate the differences between them, the data standardization method (Z-score) was adopted as the pre-processing method of the original data before using the features information for modeling and analysis. Z-score is a widely used data processing technology that can ensure the comparability of data. In addition, to reduce the dimensionality of the data, principal component analysis (PCA) was used [21].

### 2.7. Establishment of Prediction Models

Partial least squares regression (PLSR), as a classical linear model, has been widely used in tea component content prediction research [22,23,24]. The PLSR model can not only avoid the collinearity problem, but also eliminate the noise effect on regression. Support vector regression (SVR), as a nonlinear modeling method, uses a kernel function to map problems in low-dimensional space to high-dimensional space for linear regression, so as to simplify complex problems [25,26]. In this study, the radial basis kernel function (RBF) was used as the kernel function of SVR for subsequent research and analysis [27]. Moreover, extreme learning machine (ELM), as a feedforward neural network analysis method, has also shown good performance in related research [28]. Some related studies have shown that the ELM model is superior to other classical learning algorithms in terms of learning efficiency and generalization ability. Accordingly, PLS, SVR, and ELM were used to establish prediction models of tea pigments.

### 2.8. Prediction Models Evaluation Indexes

The calibration correlation coefficient (Rc), prediction coefficient (Rp), calibration root-mean-square error (RMSEC), prediction root-mean-square error (RMSEP), and residual prediction deviation (RPD) were used as evaluation indexes of the models [29,30]. In general, higher Rc, Rp, and RPD values and lower RMSEC and RMSEP values represent better model performance. Here, the data analysis was carried out using MATLAB R2021b.

## 3. Results and Discussion

### 3.1. Results of Determination of Tea Pigments Content

The variations in content of TFs, TRs, and TBs in samples at various rolling time are presented in Table 1. TFs, TRs, and TBs are oxidative polymers formed from catechins through enzymatic and hydrothermal reactions [31]. They significantly influence the color and taste of black tea. The experimental data indicate that the content of TFs, TRs, and TBs in black tea increase with prolonged rolling time. This phenomenon also explains why the color of the leaves deepens as rolling time extends. However, the rates of alteration in the content of these three substances throughout this process is not uniform. This disparity might be closely associated with the degree of fragmentation of tea tissue cells and the process of oxidative polymerization.

### 3.2. Response of Color Feature Variables During Rolling Process

**Figure 2:**
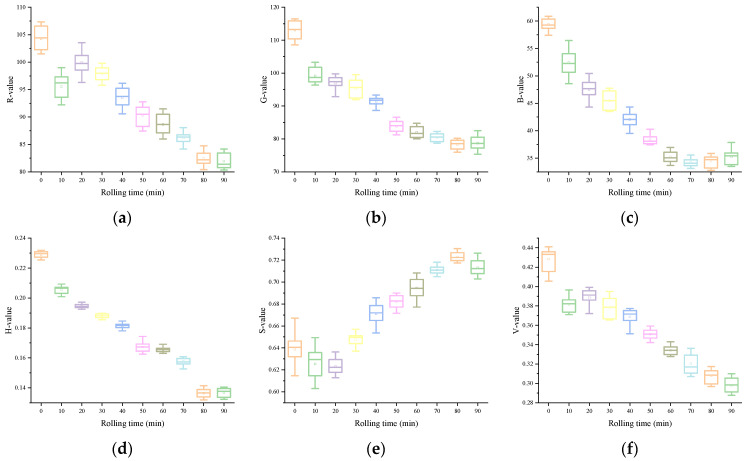

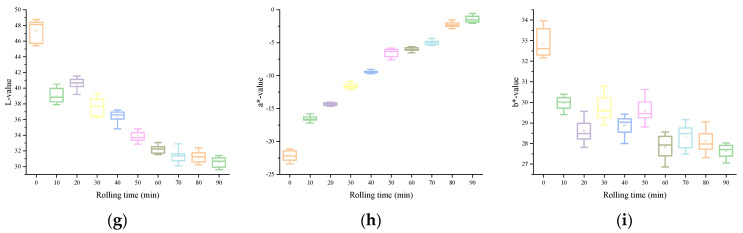
Image color feature variables: (**a**) R, (**b**) G, (**c**) B, (**d**) H, (**e**) S, (**f**) V, (**g**) L, (**h**) a*, (**i**) b*.

The variations of each color feature variable in the image information in accordance the rolling time are shown in Figure 2. From a macro point of view, the color appearance of black underwent an obvious transformation process: from green to light reddish-brown, and the brightness gradually decreases, and loses luster. In HSV, both the H and V values exhibited a gradual decline as rolling time increased, whereas the S value tended to rise consistently. This showed the appearance characteristics of the sample: with the extension of rolling time, the hue and value of the rolled leaves gradually declined. In La*b*, the changes of L and a* values were more intuitive. With a continuous increase in rolling time, L gradually decreased, and a* gradually increased. This was due to the gradual deepening and reddening appearance of the rolled leaves. However, the regularity of b* was relatively poor, which may be related to the complex interaction of multiple facets of color information during the rolling. For the RGB color model, although the R, G, and B values all showed a downward trend, the R was relatively large and the rate of decline was slower as the rolling time increased. This is also consistent with the phenomenon of the deepened red appearance of the rolled leaves. These synergistic changes of color features from different color models confirm the overall rule of color change of rolled leaves from a microscopic point of view. Through the analysis of the aforementioned color feature variables extracted from the image, it is possible to quantitatively describe the color variations occurring during the rolling process.

### 3.3. Correlation Analysis Between Color Features Variables and Tea Pigments

**Figure 3:**
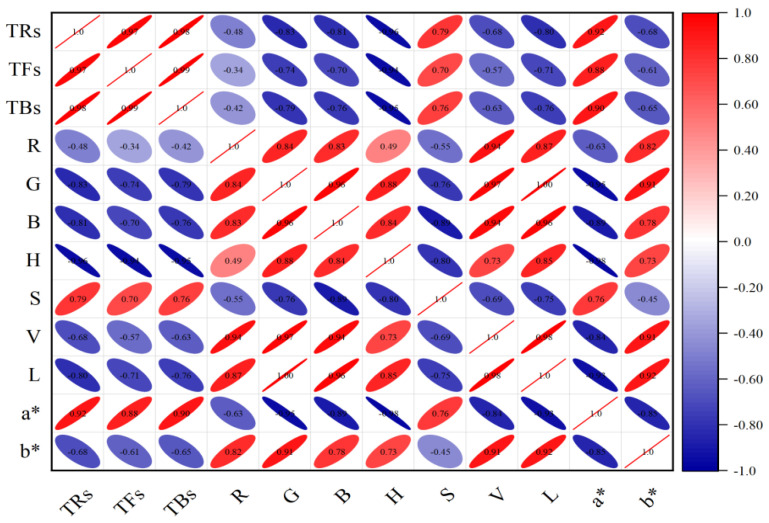
Correlation analysis diagram of tea pigments and image color feature variables.

In order to further verify the relationship between tea pigments and color variables, Pearson correlation analysis method is used in this study. It is a commonly used method to measure the correlation between variables, and its correlation coefficient results are between −1 and 1, which can effectively reveal the relationship between quantitative data. The positive and negative values of the coefficient represent positive correlation and negative correlation, respectively. The closer the result value tends to 1 and −1, the stronger the correlation is; on the contrary, the closer it tends to 0, the weaker the correlation is. The results of the correlation analysis are shown in Figure 3. The results show that the G, B, H, L, and a* values were strongly correlated with TFs. The H and a* values also demonstrated a significant correlation with TRs and TBs, where the H exhibited a negative correlation and the a* showed a positive one. The absolute values of these correlation coefficients were all greater than 0.8, showing a significant linear relationship. By comparison, the R and V values were weakly correlated with TFs, TRs, and TBs. In addition, different color feature variables also showed different degrees of correlation strength in the analysis results. Therefore, the simultaneous use of these color characteristic variables may lead to information overlap in the data, and there may be information interference among the variables, which could have a negative impact on the model’s predictive results. This further illustrates the rationality and effectiveness of selecting strongly correlated feature variables for model prediction analysis.

### 3.4. Data Preprocessing and Principal Component Analysis

**Figure 4:**
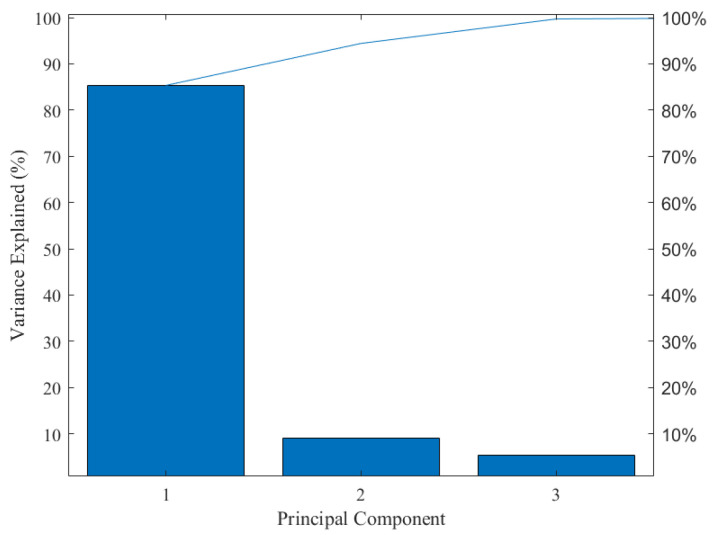
Explanatory variance in principal component analysis.

Since the color information variables in the image come from three different color models, the data information belongs to a high-dimensional array. Therefore, it is necessary to standardize the data of variables within different dimensions in order to enhance the comparability of data. Moreover, as mentioned above, some color variables are weakly correlated with tea pigments, and there are also correlations among different characteristic variables. The above situation may lead to information overlap and data redundancy. Hence, in the predictive analysis, the necessary dimensionality reduction of the data should be carried out. Based on this, Z-score is used in this study to normalize the magnitude differences among the data. Then, PCA is used to remove irrelevant variables, eliminate collinearity among the data, and reduce its dimension. The results of the PCA for color feature variables are shown in Figure 4. The explained variance for the first three principal components is 85.33%, 9.09%, and 5.29%, respectively. The cumulative contribution rate of the first three principal components is 99.71%, which reflects the effective information of the sample population.

### 3.5. Prediction Models of Tea Pigments Based on Image Information

#### 3.5.1. Prediction Results of TFs

The results of TFs prediction models based on image color feature information are shown in Table 2. Here, Z-score and Z-score–PCA were used as data preprocessing methods, respectively. These were used as model inputs, and corresponding TFs content values were used as model outputs. Meanwhile, PLSR, SVR, and ELM were used as modeling methods to establish the prediction models. The experimental data show that when the same modeling method was used, the prediction models based on Z-score–PCA data showed superior performance. This verifies that PCA can effectively eliminate redundant information in the data. Moreover, under the same data preprocessing conditions, the ELM model exhibited better performance in predicting TFs. This might be attributed to the fact that the training process of ELM is non-iterative. It initializes by randomly choosing the weight and bias of the hidden layer and subsequently directly resolves the weight of the output layer through an analytical approach. Owing to its non-iterative training mode, it can exhibit superior scalability when handling data. Consequently, the ELM will demonstrate better robustness against noise and abnormal data. In conclusion, the prediction model based on the Z-score–PCA–ELM model had the best prediction effect for TFs. Its Rc value was 0.99, RMSEC value was 0.04, Rp value was 0.97, and RMSEP value was 0.06. The RPD value reached 3.97.

**Figure 5:**
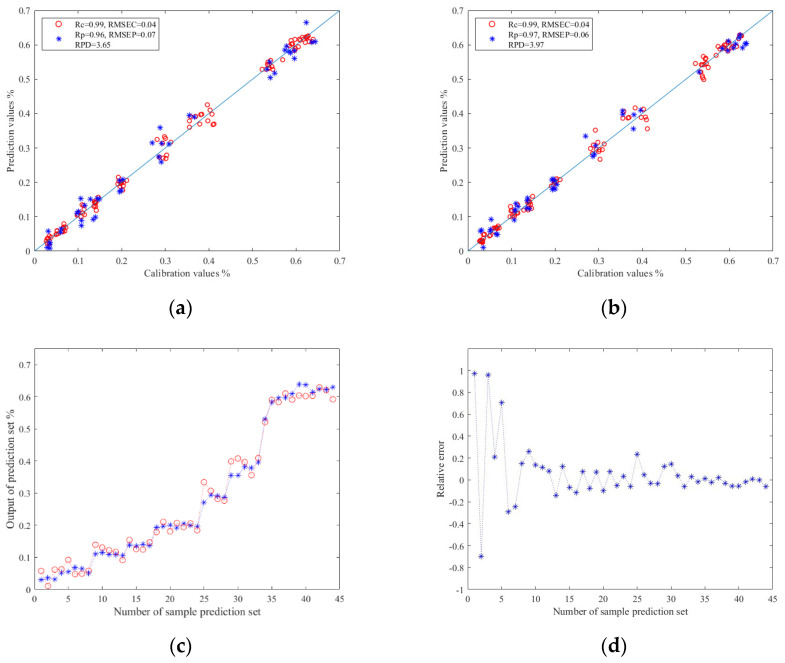
(**a**) Regression prediction scatter plot based on Z-score–ELM; (**b**) regression prediction scatter plot based on Z-score–PCA–ELM; (**c**) line chart of prediction results based on Z-score–ELM; (**d**) relative error chart of prediction results based on Z-score–PCA–ELM.

The relationship between the prediction values and calibration values in the prediction set results of the ELM models are shown in Figure 5a,b. Among them, the horizontal and vertical coordinates corresponding to the red circles represent the prediction values and calibration values of the training set samples, respectively, and the horizontal and vertical coordinates corresponding to the blue asterisks represent the prediction values and calibration values of the prediction set samples, respectively. The closer the red scatter points to the blue solid line in Figure 5a,b, the better the training effect of the model, and the closer the blue scatter points to the blue solid line, the better the prediction effect of the model.

Figure 5c,d shows the relationship diagram and relative error diagram between each correction and prediction value in the prediction set of the Z–score–PCA–ELM model, respectively. The blue asterisks represents the calibration values of the model prediction set, and the red circles represent the prediction values of the model prediction set.

#### 3.5.2. Prediction Results of TRs

**Figure 6:**
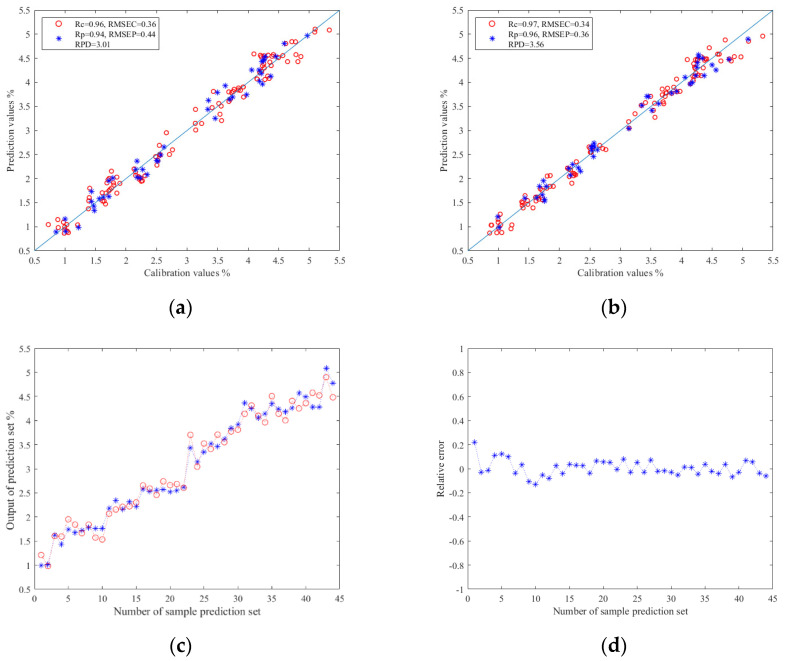
(**a**) Regression prediction scatter plot based on Z-score–ELM; (**b**) regression prediction scatter plot based on Z-score–PCA–ELM; (**c**) line chart of prediction results based on Z-score–ELM; (**d**) relative error chart of prediction results based on Z-score-PCA–ELM.

The results of the TRs prediction models based on image color feature information are shown in Table 3. As mentioned above, Z-score and Z-score–PCA were used as data preprocessing methods. PLSR, SVR, and ELM were used to construct the prediction models of TRs. The results show that when Z-score–PCA was used as the data preprocessing method, the prediction model established by ELM had the best effect: the Rc value was 0.97, the RMSEC value was 0.34, the Rp value was 0.96, the RMSEP value was 0.36, and the RPD value was 3.56. To sum up, PCA can effectively eliminate irrelevant variables between data in the prediction study of TRs using machine vision technology, and the ELM model also showed better predictability. The relationship between the prediction values and calibration values in the prediction set results of the ELM models are shown in Figure 6a,b. In addition, Figure 6c,d shows the relationship diagram and relative error diagram between each correction and prediction value in the prediction set of the Z-score–PCA–ELM model, respectively.

#### 3.5.3. Prediction Results of TBs

**Figure 7:**
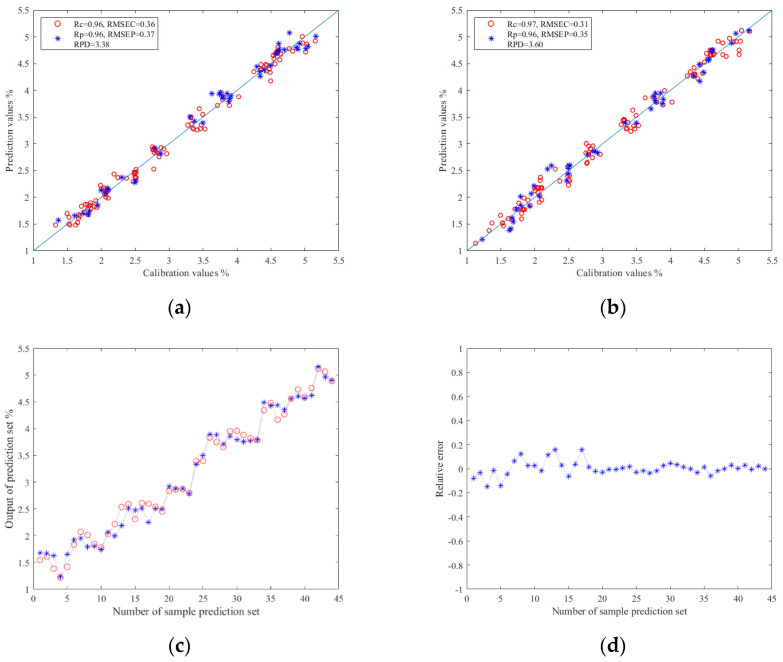
(**a**) Regression prediction scatter plot based on Z-score–ELM; (**b**) regression prediction scatter plot based on Z-score–PCA–ELM; (**c**) line chart of prediction results based on Z-score–ELM; (**d**) relative error chart of prediction results based on Z-score–PCA–ELM.

The results of TBs prediction models based on image color feature information are shown in Table 4. By comparing the experimental data, it was found that the ELM was still better than PLSR and SVR in predicting TBs. The established Z-score–PCA–ELM model had the best prediction effect: the Rc value ws 0.97, the RMSEC value was 0.31, the Rp value was 0.96, the RMSEP value was 0.35, and the RPD value reached 3.60. The results show that this model can effectively and quickly predict TBs. The relationship between the prediction values and calibration values in the prediction set results of the ELM models are shown in Figure 7a,b. Moreover, Figure 7c,d shows the relationship diagram and relative error diagram between each correction and prediction value in the prediction set of the Z-score–PCA–ELM model, respectively. They provide an intuitive display of the results of the prediction model.

## 4. Conclusions

In this article, machine vision technology is employed to establish a rapid detection method for tea pigments during the rolling of black tea. The variation rules of color feature variables in the rolling process are discussed, and the correlation between color variables and tea pigments is characterized. The Z-score and Z-score–PCA are used as preprocessing techniques, which are subsequently combined with PLSR, SVR, and ELM for comparative analysis. The results show that Z-score–PCA is more advantageous for the application analysis of color feature variables. Meanwhile, ELM shows superior prediction accuracy. The Z-score–PCA–ELM model established in this study has the best prediction results for TFs, TRs, and TBs. This indicates that by combining machine vision technology with the machine learning algorithms, it is possible to quickly detect the content of tea pigments during rolling process. This study establishes innovative methods for the online evaluation of the rolling process in the high-efficiency industrial production of black tea.

## Figures and Tables

**Table 1:** Changes in tea pigments content during rolling of black tea.

Rolling Time (min)	Number of Samples	TFs (%)	TRs (%)	TBs (%)
0	15	0.03 ± 0.00	0.99 ± 0.12	1.52 ± 0.19
10	15	0.06 ± 0.01	1.52 ± 0.11	1.83 ± 0.07
20	15	0.11 ± 0.01	1.76 ± 0.07	2.06 ± 0.04
30	15	0.14 ± 0.01	2.22 ± 0.06	2.44 ± 0.11
40	15	0.20 ± 0.01	2.57 ± 0.08	2.83 ± 0.06
50	15	0.29 ± 0.11	3.41 ± 0.19	3.40 ± 0.08
60	15	0.38 ± 0.02	3.79 ± 0.13	3.82 ± 0.10
70	15	0.54 ± 0.01	4.23 ± 0.08	4.40 ± 0.08
80	15	0.59 ± 0.01	4.32 ± 0.11	4.60 ± 0.03
90	15	0.63 ± 0.01	4.75 ± 0.31	4.94 ± 0.14

**Table 2:** Results of theaflavins (TFs) prediction models based on machine vision technology.

Types of Models	Pretreatment Methods	Training Set		Prediction Set		
		Rc	RMSEC	Rp	RMSEP	RPD
PLSR	Z-score	0.93	0.08	0.91	0.12	2.01
	Z-score–PCA	0.94	0.07	0.94	0.10	2.19
SVR	Z-score	0.98	0.05	0.94	0.09	2.17
	Z-score–PCA	0.95	0.07	0.95	0.07	2.67
ELM	Z-score	0.99	0.04	0.96	0.07	3.65
	Z-score–PCA	0.99	0.04	0.97	0.06	3.97

**Table 3:** Results of thearubigins (TRs) prediction models based on machine vision technology.

Types of Models	Pretreatment Methods	Training Set		Prediction Set		
		Rc	RMSEC	Rp	RMSEP	RPD
PLSR	Z-score	0.96	0.35	0.93	0.51	2.35
	Z-score-PCA	0.95	0.41	0.94	0.44	2.71
SVR	Z-score	0.96	0.34	0.94	0.50	2.56
	Z-score-PCA	0.96	0.35	0.95	0.44	2.73
ELM	Z-score	0.96	0.36	0.94	0.44	3.01
	Z-score-PCA	0.97	0.34	0.96	0.36	3.56

**Table 4:** Results of theabrownins (TBs) prediction models based on machine vision technology.

Types of Models	Pretreatment Methods	Training Set		Prediction Set		
		Rc	RMSEC	Rp	RMSEP	RPD
PLSR	Z-score	0.96	0.34	0.94	0.51	2.08
	Z-score–PCA	0.94	0.38	0.94	0.41	2.45
SVR	Z-score	0.97	0.32	0.94	0.40	2.82
	Z-score–PCA	0.95	0.37	0.95	0.37	3.02
ELM	Z-score	0.96	0.36	0.96	0.37	3.38
	Z-score–PCA	0.97	0.31	0.96	0.35	3.60

## Data Availability

The original contributions presented in this study are included in the article. Further inquiries can be directed to the corresponding author.

## References

[1] Chen Q.C., Fu Y., Heng W.T., Yu S., Xie F., Dong F., Lin Z., Dai W.D., Fu H.H. (2024). Re-rolling treatment in the fermentation process improves the taste and liquor color qualities of black tea. Food Chem.-X.

[2] Wang A.L., Lei Q.Q., Zhang B.B., Wu J.H., Fu Z.Y., He J.F., Wang Y.B., Wu X.Y. (2024). Revealing novel insights into the enhancement of quality in black tea processing through microbial intervention. Food Chem.-X.

[3] Wu S.M., Yu Q.Y., Shen S., Shan X.J., Hua J.J., Zhu J.Y., Qiu J.R., Deng Y.L., Zhou Q.H., Jiang Y.W. (2022). Non-targeted metabolomics and electronic tongue analysis reveal the effect of rolling time on the sensory quality and nonvolatile metabolites of congou black tea. Lwt-Food Sci. Technol..

[4] Zhang S., Wu S.M., Yu Q.Y., Shan X.J., Chen L., Deng Y.L., Hua J.J., Zhu J.Y., Zhou Q.H., Jiang Y.W. (2023). The influence of rolling pressure on the changes in non-volatile compounds and sensory quality of congou black tea: The combination of metabolomics, E-tongue, and chromatic differences analyses. Food Chem.-X.

[5] Hua J.J., Xu Q., Yuan H.B., Wang J.J., Wu Z.Q., Li X.T., Jiang Y.W. (2021). Effects of novel fermentation method on the biochemical components change and quality formation of Congou black tea. J. Food Compos. Anal..

[6] Liu M., Wang R.X., Shi D.L., Cao R.Y. (2024). Non-destructive prediction of tea polyphenols during Pu-erh tea fermentation using NIR coupled with chemometrics methods. J. Food Compos. Anal..

[7] Turgut S.S., Entrenas J.A., Taskin E., Garrido-Varo A., Pérez-Marín D. (2022). Estimation of the sensory properties of black tea samples using non-destructive near-infrared spectroscopy sensors. Food Control.

[8] Luo W., Tian P., Fan G.Z., Dong W.T., Zhang H.L., Liu X.M. (2022). Non-destructive determination of four tea polyphenols in fresh tea using visible and near-infrared spectroscopy. Infrared Phys. Technol..

[9] Wen J., Xu G.Q., Zhang A., Ma W., Jin G. (2024). Emerging technologies for rapid non-destructive testing of grape quality: A review. J. Food Compos. Anal..

[10] Jiang Y.Y., Zhang D.X., Yang L., Cui T., He X.T., Wu D.Y., Dong J.Q., Li C., Xing S.L. (2024). Design and experiment of non-destructive testing system for moisture content of in-situ maize ear kernels based on VIS-NIR. J. Food Compos. Anal..

[11] Maniwara P., Nakano K., Ohashi S., Boonyakiat D., Seehanam P., Theanjumpo P., Poonlarp P. (2019). Evaluation of NIRS as non-destructive test to evaluate quality traits of purple passion fruit. Sci. Hortic..

[12] Wei Y., Wen Y.Q., Huang X.L., Ma P.H., Wang L., Pan Y., Lv Y.J., Wang H.X., Zhang L., Wang K.B. (2024). The dawn of intelligent technologies in tea industry. Trends Food Sci. Technol..

[13] Wang Y.J., Ren Z.Y., Chen Y.Y., Lu C.Y., Deng W.W., Zhang Z.Z., Ning J.M. (2023). Visualizing chemical indicators: Spatial and temporal quality formation and distribution during black tea fermentation. Food Chem..

[14] Zhu H.K., Liu F., Ye Y., Chen L., Li J.Y., Gu A.H., Zhang J.Q., Dong C.W. (2019). Application of machine learning algorithms in quality assurance of fermentation process of black tea- based on electrical properties. J. Food Eng..

[15] Yao L.H., Jiang Y.M., Caffin N., D’Arcy B., Datta N., Liu X., Singanusong R., Xu Y. (2006). Phenolic compounds in tea from Australian supermarkets. Food Chem..

[16] Ouyang Q., Wang L., Park B., Kang R., Chen Q.S. (2021). Simultaneous quantification of chemical constituents in matcha with visible-near infrared hyperspectral imaging technology. Food Chem..

[17] Chemov V., Alander J., Bochko V. (2015). Integer-based accurate conversion between RGB and HSV color spaces. Comput. Electr. Eng..

[18] Paschos G. (2001). Perceptually uniform color spaces for color texture analysis: An empirical evaluation. IEEE Trans. Image Process..

[19] Lan T.M., Shen S., Yuan H.B., Jiang Y.W., Tong H.R., Ye Y. (2022). A Rapid Prediction Method of Moisture Content for Green Tea Fixation Based on WOA-Elman. Foods.

[20] Shen S., Hua J.J., Zhu H.K., Yang Y.Q., Deng Y.L., Li J., Yuan H.B., Wang J.J., Zhu J.Y., Jiang Y.W. (2022). Rapid and real-time detection of moisture in black tea during withering using micro-near-infrared spectroscopy. Lwt-Food Sci. Technol..

[21] Zheng L., Watson D.G., Johnston B.F., Clark R.L., Edrada-Ebel R., Elseheri W. (2009). A chemometric study of chromatograms of tea extracts by correlation optimization warping in conjunction with PCA, support vector machines and random forest data modeling. Anal. Chim. Acta.

[22] Asante E.A., Du Z., Lu Y.Z., Hu Y.G. (2021). Detection and assessment of nitrogen effect on cold tolerance for tea by hyperspectral reflectance with PLSR, PCR, and LM models. Inf. Process. Agric..

[23] Amsaraj R., Mutturi S. (2021). Real-coded GA coupled to PLS for rapid detection and quantification of tartrazine in tea using FT-IR spectroscopy. Lwt-Food Sci. Technol..

[24] Sobhaninia M., Mani-Varnosfaderani A., Barzegar M., Sahari M.A. (2024). Combining ion mobility spectrometry and chemometrics for detecting synthetic colorants in black tea: A reliable and fast method. Food Chem.-X.

[25] Xie G., Qian Y.T., Wang S.Y. (2021). Forecasting Chinese cruise tourism demand with big data: An optimized machine learning approach. Tour. Manag..

[26] Wei X., Li S., Zhu S.P., Zheng W.Q., Xie Y., Zhou S.L., Hu M.D., Miao Y.J., Ma L.K., Wu W.J. (2021). Terahertz spectroscopy combined with data dimensionality reduction algorithms for quantitative analysis of protein content in soybeans. Spectrochim. Acta Part A-Mol. Biomol. Spectrosc..

[27] Yang Y.C., Sun D.W., Wang N.N. (2015). Rapid detection of browning levels of lychee pericarp as affected by moisture contents using hyperspectral imaging. Comput. Electron. Agric..

[28] Li H., Hu Y., Ma S., Haruna S.A., Chen Q., Zhu W., Xia A. (2024). Porphyrin and pH sensitive dye-based colorimetric sensor array coupled chemometrics for dynamic monitoring of tea quality during ultrasound-assisted fermentation. Microchem. J..

[29] Ren G.X., Yin L.L., Wu R., Ning J.M. (2024). Rapid detection of ash content in black tea using a homemade miniature near-infrared spectroscopy. Spectrochim. Acta Part A-Mol. Biomol. Spectrosc..

[30] Dai F.S., Shi J., Yang C.S., Li Y., Zhao Y., Liu Z.Y., An T., Li X.L., Yan P., Dong C.W. (2023). Detection of anthocyanin content in fresh Zijuan tea leaves based on hyperspectral imaging. Food Control.

[31] Zhang L., Ho C.T., Zhou J., Santos J.S., Armstrong L., Granato D. (2019). Chemistry and Biological Activities of Processed Camellia sinensis Teas: A Comprehensive Review. Compr. Rev. Food Sci. Food Saf..

